# A Practical Deep Learning Model in Differentiating Pneumonia-Type Lung Carcinoma from Pneumonia on CT Images: ResNet Added with Attention Mechanism

**DOI:** 10.1155/2022/8906259

**Published:** 2022-02-23

**Authors:** Wang Du, Xiaojie Luo, Min Chen

**Affiliations:** Department of Radiology, Beijing Hospital, National Center of Gerontology, Institute of Geriatric Medicine, Chinese Academy of Medical Science, Beijing 100730, China

## Abstract

**Objective:**

We aim to develop a deep neural network model to differentiate pneumonia-type lung carcinoma from pneumonia based on chest CT scanning and evaluate its performance.

**Materials and Methods:**

We retrospectively analyzed 131 patients diagnosed with pneumonia-type lung carcinoma and 171 patients with pneumonia treated in Beijing Hospital from October 2019 to February 2021. The average age was 68 (±15) years old, and the proportion of men (162/302) was slightly more than that of women (140/302). In this study, a deep learning based model UNet was applied to extract lesion areas from chest CT images. Lesion areas were extracted and classified by a designed spatial attention mechanism network. The model AUC and diagnostic accuracy were analyzed based on the results of the model. We analyzed the accuracy rate, sensitivity, and specificity and compared the results of the model to the junior and senior radiologists and radiologists based on the model.

**Results:**

The model has a good efficiency in detecting pneumonia-like lesions (6.31 seconds/case). The model accuracy rate, sensitivity, and specificity were 74.20%, 60.37%, and 89.36%, respectively. The junior radiologist's accuracy rate, sensitivity, and specificity were 61.00%, 48.08%, and 75.00%, respectively. The senior radiologist's accuracy rate, sensitivity, and specificity were 65.00%, 51.92%, and 79.17%, respectively. The results of junior radiologists based on the model were improved (76.00% for accuracy rate, 62.75% for sensitivity, and 89.80% for specificity). The results of senior radiologists based on the model were also improved (78.00% for accuracy rate, 64.71% for sensitivity, and 91.84% for specificity) and the diagnostic accuracy of which was statistically higher than other groups (*P* < 0.05). Based on the lesion texture diversity and the lesion boundary ambiguity, the algorithm produced false-positive samples (13.51%).

**Conclusion:**

This deep learning model could detect pneumonia-type lung carcinoma and differentiate it from pneumonia accurately and efficiently.

## 1. Introduction

Lung cancer is the most common malignant tumor in the pulmonary disease, with about 1.8 million patients worldwide [1]. The incidence has increased significantly in the last 50 years [2]. Lung cancer is clinically divided into small cell lung cancer (SCLC) and nonsmall cell lung cancer (NSCLC), among which NSCLC accounts for 80%. The most common histological subtypes of NSCLC are adenocarcinoma and squamous cell carcinoma (SQCC) [3]. However, in recent years, the incidence of pneumonic type lung carcinoma (PTLC) has been increasing, accounting for 0.48–3.33% of primary lung cancer, which is mainly seen in middle-aged and elderly patients, and the pathological type is adenocarcinoma [4,5]. Most patients with PTLC have a history of smoking, with the main manifestations of cough and sputum accompanied by fever. Most of the lesions are distributed around the lung. Laboratory and imaging examinations show no characteristic manifestations in the early stage of the disease, resulting in a high rate of misdiagnoses as pneumonia, tuberculosis, among other diseases in terms of symptoms and imaging manifestations at the first diagnosis [6–8].

Recently, computed tomography has been continuously developing. HRCT, the MPR function of MSCT, energy spectrum CT quantitative analysis, and dual-source CT dual-energy technology have been applied in the differential diagnosis of PTLC and pneumonia. They provided more information for CT diagnosis, thus enlarged the application scope of CT and improved diagnostic accuracy. Meanwhile, deep learning technology has achieved great success in medical imaging due to the powerful feature extraction ability [9–11]. Specifically, deep learning has been applied to children's chest radiograms to detect and identify bacterial and viral pneumonia [12, 13]. There have also been attempts to detect various imaging features of chest CT [14, 15]. At present, studies have applied deep learning technology for detecting and analyzing pneumonia lesions in CT images [16–18]. Other studies conducted an algorithmic analysis of lung cancer [19–21]. However, the current work mainly focuses on analyzing disease separately. There is a lack of study on the differentiation of multiple diseases, especially pneumonia and PTLC, which is very similar in imaging characteristics. Therefore, specific models were warranted to distinguish between the two diseases. The ResNet network uses cross-layer connections to strengthen the ability of gradient backpropagation, and attention mechanism is commonly used as an explicit feature selection operation in the field of computer vision. We used the RESNET neural network and added the attention mechanism to accurately locate the features in the network and extract the critical features for diagnosis. CT images of pneumonia and PTLC were included to test the stability of the model.

## 2. Materials and Methods

### 2.1. Patients

The institutional ethics committee approved this retrospective study. The need to obtain written informed consent for participation was waived.

Chest CT images from 302 patients from October 2019 to February 2021 were included in this study, comprising 171 patients with pneumonia and 131 patients with PTLC. Among them, there were 162 males and 140 females with an average age of 68.

Inclusion criteria: patients with pneumonia-type lung cancer or pneumonia diagnosed by CT were included. The image quality of CT was good. Exclusion criteria: cases with poor image quality affecting observation.

All eligible patients underwent laboratory examination, routine pathological examination, or anti-inflammatory therapy.

The chest CT image with pneumonia and PTLC were randomly selected from October 2019 to February 2021 and randomly divided into a training set and a test set at a ratio of 2 : 1. The training dataset was then further subdivided into the training and internal validation (10% of the sample). Independent test sets were not used for training and internal validation. The patient demographic and disease statistics are given in Tables 1 and 2, separately.

### 2.2. Imaging Protocol

The chest CT image was obtained with equipment from different manufacturers by using standard imaging protocols. Chest CT scan field from the thoracic inlet to the base of the lungs. Protocols are as follows: scanning slice thickness, 5 mm; image matrix, 512 × 512; FOV, 400 mm; reconstruction parameter setting: slice thickness, 1.0 mm; layer spacing, 1.0 mm; observation window parameters: lung window width, 1500 HU, window position, −600 HU; mediastinum window width, 400 HU; window level, 35 HU.

### 2.3. Deep Learning Model

**Figure 1:**
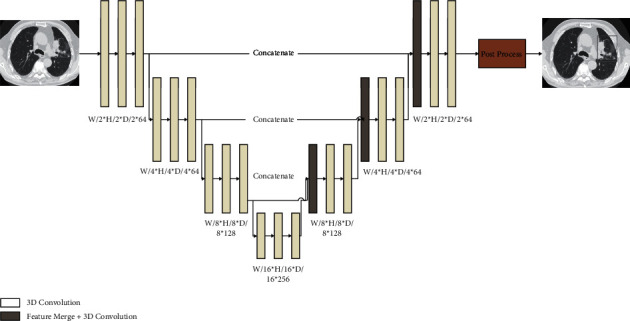
Segmentation model based on UNet.

First, a segmentation model based on UNet [22] was designed (Figure 1). UNet employed feature extraction and reconstruction are based on multiscale information fusion. Specifically, the extraction model was a cascade of residual block [23] to extract CT image feature from different scales. Then, UNet performed feature reconstruction. When the feature scaled up, the features of previous reconstruction and corresponding scale in the downsampling process were merged to ensure the completeness of the information.

**Figure 2:**
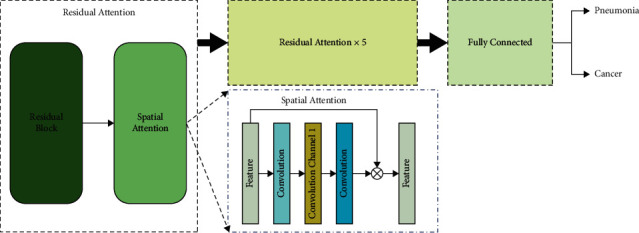
Architecture for the lesion classification.

In the stage of lesion classification, we designed a classification network with the spatial attention mechanism (Figure 2). In the process of feature extraction, the attention mechanism was used to filter out unrelated features. The spatial attention network is a ResNet network [23]. Before each feature downsampling, the features were fed to a convolution module with a constant output channel of 1, and the sigmoid function was applied to the output to generate a weighted mask with the same width as feature. The value of each element in the weight mask ranged from 0 to 1, which was multiplied by the feature to complete the feature filtering. The UNet model achieved the effect of state of the art in the field of semantic segmentation due to its structure of multilevel feature reconstruction and reuse of downsampling features. Meanwhile, UNet's multilevel feature multiplexing in the process of feature encoding greatly reduced the information loss in the network information transmission. Thus, the network had a better segmentation effect in difficult areas such as boundaries.

The attention mechanism made the network focus on important parts of the image during feature extraction. The information contained in the image was rich and diverse. The indiscriminate reference of all the information in the image would make the features complicated and confusing. The attention mechanism ensured that only the key features in the image were extracted by assigning different weights to the image features of different regions, resulting in decreased cost of network convergence and improved accuracy.

### 2.4. Detection of Pneumonia-Like Lesions

#### 2.4.1. Artificial Diagnosis

A junior radiologist with 3–5 years of work experience and a senior chest imaging specialist with more than ten years of work experience independently read out the images without referring to the deep learning model. The location of pneumonia-like lesions was marked; the lesion features were selected; the qualitative diagnosis was made; the number of image layers was recorded based on the location of the lesions.

#### 2.4.2. The Deep Learning Model Detected Pneumonia-Like Lesions

The chest CT images of 100 patients were imported into the model for automatic recognition, labeling, and diagnosis.

#### 2.4.3. Deep Learning Models Assisted Radiologists in Diagnosis

Two weeks after the radiologist completed the independent readout of the images, the junior and senior radiologists used the deep learning model to assist the reading and recorded the diagnostic accuracy of pneumonia-like lesions detected.

#### 2.4.4. Reference Criteria for Qualitative Diagnosis of Pneumonia-Like Lesions

Two chest radiologists with more than ten years of experience, respectively, marked the chest CT images of 302 patients. The diagnostic results of the lesion were identified and recorded. The consensual results were combined with the results of pathology and laboratory tests as the reference standard for genuine positivity.

### 2.5. Statistical Analysis

SPSS 20.0 (IBM Corp., Armonk, NY, USA) was used to input, organize, and analyze the data. Levels of significant are shown as *P* < 0.05.

1. The performance of our trained deep learning model was evaluated using an independent test set that was not involved in the training process. The test set consisted of 100 examination images, which were randomly selected from 302 data. Unrestricted response receiver operating characteristic (ROC) curve was used to analyze and test the performance of the deep learning model to comprehensively reflect the performance of the model in terms of false-positive rate and detection rate. The area under the curve was calculated using a 95% confidence interval (CI).
2. With the assistance of the deep learning model, the differences in the diagnostic accuracy of pneumonia-like lesions among the five groups were compared among the doctors with low seniority, the doctors with high seniority, the deep learning model, and the combined deep learning model of the doctors with high and low seniority, respectively. The chi-square test was used to make pairwise comparison between groups.

## 3. Results

### 3.1. Study Population Characteristics

Table 1 provides the demographic characteristics for the training and independent testing datasets. There were slightly more male than female patients across PTLC and pneumonia groups for both the training dataset (percentage of male patients: PTLC 19.8%; pneumonia, 33.7%; *P*=0.55) and the independent testing dataset (percentage of male patients: PTLC 27%; pneumonia, 33%; *P*=0.468). There was no significant difference in age between the two groups.

### 3.2. Deep Learning Model Performance

**Figure 3:**
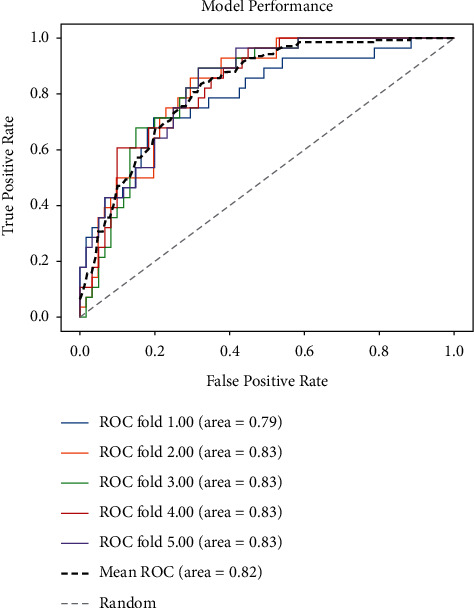
5-fold validation ROC curves: the black dash line means the average of 5 curves, and the gray dash line means a model without any predictive ability. The average AUC value of FROC was 0.82 (*P* < 0.01) after five-fold cross-validation. The average accuracy of cross-validation was 74.2%. The model introduced false-positive in the process of distinguishing pneumonia and lung cancer, but the overall accuracy had a relatively strong reference value. It shows that the model has high credibility.

**Figure 4:**
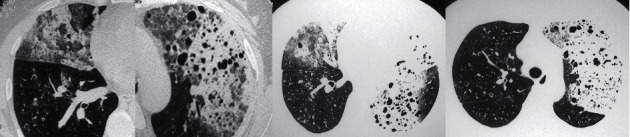
Representative example of a case of pneumonia-type lung carcinoma misclassified as pneumonia. The different slices around the abnormality are shown.

**Figure 5:**
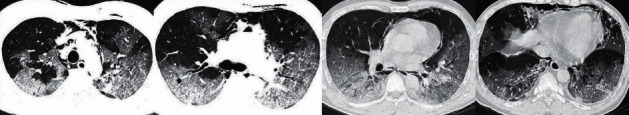
Representative example of a case of pneumonia misclassified as pneumonia-type lung carcinoma. The different slices around the abnormality are shown.

Once the model was trained, it was deployed on a server with NVIDIA 2080Ti graphics card, 16 GB RAM, Intel Xeon E5-2620 v4 @2.10 GHz. Inference speed of the model on this platform was fast, 6.25 seconds per study on average. The detailed performance of the model is shown in figure. An average AUC value of 0.82 (*P* < 0.01) (Figure 3) was obtained via five-fold cross-validation. In the validation set, 74 cases were correctly diagnosed with pneumonia-like lesions and 26 cases were diagnosed incorrectly (Figures 4 and 5). The average accuracy of five-fold cross-validation was 74.2% (Table 2). The model introduces false-positives in the process of distinguishing PTLC from pneumonia. The overall accuracy rate has a relatively strong reference value, but some cases seemed to be very challenging cases. In order to further improve the accuracy, more data might be required for multiple training.

### 3.3. Comparison of Diagnostic Accuracy between the Deep Learning Model and Radiologists for Pneumonia-Like Lesions

**Figure 6:**
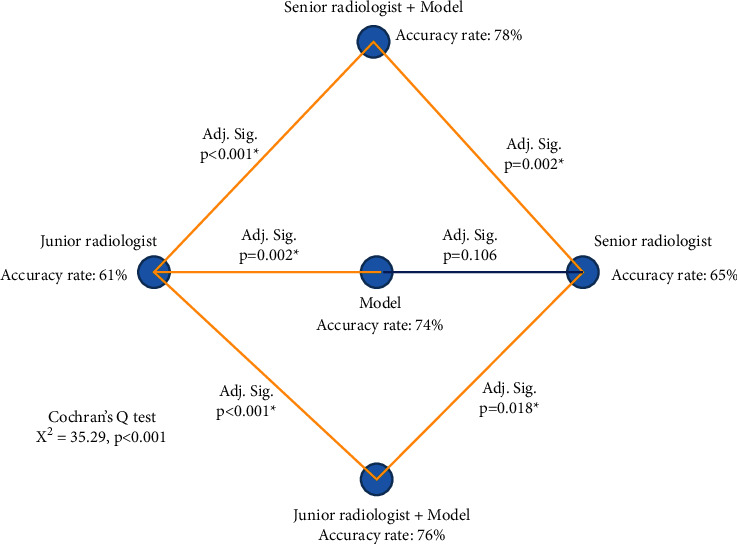
Comparison of overall diagnostic accuracy of pneumonia-like lesions among junior radiologist, senior radiologist, model, and radiologist joint model. Adj. Sig, adjusted significance. ^*∗*^Variables significantly different (*P* < 0.05).

- The details of junior radiologists, senior radiologists, deep learning model, and radiologist based on the model of overall reading pneumonia sample number of correct diagnosis, correct diagnosis score (lesion localization fraction, LLF), false-positive diagnostic score (nonlesion localization fraction, NLF), sensitivity, specific, positive predictive value, and negative predictive value are given in Table 2.
- Comparison of diagnostic accuracy of pneumonia-like disease among junior radiologists, senior radiologists, model, and radiologist based on model (Figure 6). The diagnostic accuracy of senior radiologists based on the model was the highest, which was statistically different from other groups (*P* < 0.05). The corresponding diagnostic accuracy rates of each group were 61%, 65%, 74%, 76%, and 78%, respectively, as given in Table 2. Results of pairwise comparison between groups are given in Table 2. There were no statistically significant differences in the diagnostic accuracy between any two following groups, including senior and junior radiologists (*P*=0.558), model and senior radiologists (*P*=0.167), model and senior radiologist based on the model (*P*=0.774), model and junior radiologist based on model (*P*=0.508), and junior radiologist based on model and senior radiologist (*P*=0.088). There were statistically significant differences in the diagnostic accuracy between any two following groups, including the model and the junior radiologist, the junior radiologist based on the model and the junior radiologist, the senior radiologist based on the model and the junior radiologist, and the senior radiologist based on model and the senior radiologist (all *P* < 0.05).

## 4. Discussion

PTLC is a special manifestation of peripheral lung cancer, which mainly manifested as lung lobe, segment, and ground glass density in imaging finding. The clinical manifestations are cough, expectoration, fever, and other symptoms, lack of specificity, with similar imaging to “pneumonia,” which can be easily misdiagnosed as pneumonia and other diseases. As a result, patients cannot receive effective treatment in the first time, directly threatening the follow-up treatment and life [22–24]. Clinically, most patients with PTLC have pneumonia-like imaging changes [25], and mucinous cell type mucinous adenocarcinoma is the main pathological type [26]. In the past, PTLC was often assessed according to the imaging manifestations of bronchus accompanied by branch vessels, and it could be considered as PTLC in the case of pneumonia accompanied by irregular deformation and stenosis of bronchial lumen. Misdiagnosis or missed diagnosis often occurred in conventional CT examination [27].

In recent years, imaging technology in China has developed rapidly, and high-precision CT and PET/CT have become increasingly popular, which is of great help to distinguish between PTLC with different pathological basis and focal pneumonia. In a study, patients with pneumonia-type lung cancer received chest-enhanced CT, single-phase PET/CT, and dual-phase 18F-FDG PET/CT, respectively. The study found that chest enhanced CT combined with dual-phase F-FDG PET/CT had the highest diagnostic accuracy (91.1%). The accuracy of chest enhanced CT alone was the lowest (27.4%), proving that chest enhanced CT combined with dual-phase 18F-FDG PET/CT has an exact diagnostic effect. However, it is still misdiagnosed to a certain extent to assess benign and malignant lesions based on the 18F-FDG glucose metabolism [28]. This examination will increase the patient's economic burden and radiation dose. In the past, when distinguishing PTLC from pneumonia, biopsy and pathology were used in addition to conventional CT examination, so as to promote the improvement of the diagnosis effect of PTLC. When using fibrobronchoscopy histological biopsy, the pathological diagnosis can be carried out under the guidance of CT, which can not only accurately judge the benign and malignant tumor but also clearly classify the tumor, providing a good diagnostic basis for subsequent treatment. The PTLC lacks the irregular mass and burr signs common to lung cancer on imaging, but is actually composed of numerous cancerous nodules without inflammatory lesions. The pathological basis may be due to the invasive development of cancer tissue itself in bronchus and alveoli, which covers the surface of alveolar wall when spreading in the airway and leads to consolidation of lung lobe in the process of mucus secretion [29]. However, there are some limitations in the puncture biopsy. Due to the influence of small biopsy specimens and deviation of sample acquisition site, there are sometimes missed diagnosis and misdiagnosis. Moreover, puncture biopsy is an invasive examination, which will bring psychological burden to patients.

With the rise of machine learning in recent years, some works have been focused on the field of pneumonia image processing. For example, a convolutional neural network was used to distinguish between pneumonia patients and healthy controls [30]. Besides, novel coronavirus pneumonia was differentiated from general pneumonia using the convolutional neural network [31]. Moreover, a variety of deep learning feature embedding methods were used to improve the recognition accuracy of COVID-19 pneumonia [32], screen diseased frames from pneumonia images [33], and screen patients with pneumonia [34]. However, there is little work in the field of distinguishing pneumonia and lung cancer. UNet achieves excellent results in the field of medical image segmentation because of its excellent feature coding structure [35–38]. The ResNet network uses cross-layer connections to strengthen the ability of gradient backpropagation, which is regarded as the best backbone network in many image processing tasks [39–41]. Attention Mechanism, as a commonly used explicit feature selection operation in the field of computer vision, affects the development of many machine learning tasks [42–45]. We have proposed a new model for the identification of PTLC and pneumonia and achieved good results via the combination of these technologies. In this study, we designed and evaluated a deep learning model to differentiate PTLC and pneumonia from the chest CT image. On an independent testing dataset, we showed that this model has high sensitivity (60.37%) and specificity (89.36%) in the detection of PTLC and pneumonia. The areas under the receiver operating characteristic curves for the model was 0.82, suggesting good performance and high credibility. In terms of diagnostic accuracy, the combined model of senior radiologists was the highest (78%). The model was higher than the manual model of junior radiologists and had no significant difference with the combined model of radiologists. This study showed that the sensitivity of the diagnosis increased from 48.08% to 64.71%, the specificity increased from 75.00% to 91.84%, and the false-positive rate decreased from 32.43% to 10.81% after the radiologists used the model. Therefore, the application of the model improved the diagnostic accuracy and efficiency.

However, this study has some limitations. There is a lot of overlap in the response of the lungs to various injuries, as well as in the presentation of many lung diseases. There is no way to distinguish all lung diseases based solely on the appearance of the chest CT image. At present, it is generally believed at home and abroad that the final results of computer-aided diagnosis should still be confirmed by the radiologists, so we suggest a multidisciplinary approach. In addition, although a large amount of data were collected for this study, the test set and the training set came from the same hospital. Finally, there is still room for improvement in the encoding of features. The feature extraction method based on convolution cannot pay attention to the global feature distribution of the lesion. The transformer [46] structure commonly used in the ViT field can be used to enhance the feature encoding to strengthen the attention of the feature to the global information of the lesion.

In conclusion, a machine learning method based on the convolutional network model was used to distinguish PTLC and pneumonia in this study. The results showed that the model has good performance and high reliability, and the diagnostic accuracy is significantly higher than simple chest CT. Meanwhile, compared with traditional examination methods, this method reduce radiation, economic, and psychological burden for patients. Therefore, this method has prospect for clinical application. However, this model has some deficiencies. In the future, we will collect more cases, increase the sample size, subdivide the pathological types, optimize the model, and apply the updated algorithm. We hope to develop a mature deep learning model to differentiate PTLC and pneumonia from the chest CT image more accurately and provide reference for treatment.

## Figures and Tables

**Table 1:** Summary of training and independent testing datasets.

Training set			Independent testing set		
Parameter	PTLC		Pneumonia	PTLC		Pneumonia
	*P* value			*P* value		
No. of patients	88 (43.6)	114 (56.4)		43 (43)	57 (57)	
Male patients	40 (19.8)	68 (33.7)	0.550	27 (27.0)	33 (33.0)	0.468
Age (*y*) ^*∗*^	68.23 ± 12.46	66.28 ± 16.16	0.347	69.42 ± 16.54	69.34 ± 15.21	0.424
Pathology examination	Adenocarcinoma, *n* = 67; squamous cell carcinomas, *n* = 14; small cell undifferentiated carcinoma, *n* = 7	Bacterial pneumonia, *n* = 24; viral pneumonia, *n* = 11; CAP, *n* = 79		Adenocarcinoma, *n* = 33; squamous cell carcinomas, *n* = 7; small cell undifferentiated carcinoma, *n* = 3	Bacterial pneumonia, *n* = 16; viral pneumonia, *n* = 7; CAP, *n* = 34	

Values in parentheses are percentages. PTLC, pneumonic-type lung carcinoma; CAP, community-acquired pneumonia. ^*∗*^Ages are reported as means ± standard deviations.

**Table 2:** Evaluation indexes of overall effectiveness of the radiologist, deep learning, and radiologist joint model in diagnosing pneumonia-like lesions.

	Junior radiologist	Senior radiologist	Model	Junior radiologist + model	Senior radiologist + model
No. of correct diagnosis	25/36	27/38	32/42	32/44	33/45
LLF	61%	65%	74%	76%	78%
NLF	32.4% (95% CI: 0.19–0.50)	27.01% (95% CI: 0.14–0.44)	13.51% (95% CI: 0.05–0.30)	13.51% (95% CI: 0.05–0.30)	10.81% (95% CI: 0.04–0.26)
Sensitivity	48.0% (95% CI: 0.34–0.62)	51.92% (95% CI: 0.38–0.66)	60.37% (95% CI: 0.46–0.73)	62.75% (95% CI: 0.48–0.76)	64.71% (95% CI: 0.50–0.77)
Specificity	75.0% (95% CI: 0.60–0.86)	79.17% (95% CI: 0.65–0.89)	89.36% (95% CI: 0.76–0.96)	89.80% (95% CI: 0.77–0.96)	91.84% (95% CI: 0.80–0.97)
PPV	67.5% (95% CI: 0.50–0.81)	72.97% (95% CI: 0.56–0.86)	86.49% (95% CI: 0.70–0.95)	86.49% (95% CI: 0.70–0.95)	89.19% (95% CI: 0.74–0.96)
NPV	57.1% (95% CI: 0.44–0.69)	60.31% (95% CI: 0.47–0.72)	66.67% (95% CI: 0.54–0.78)	69.84% (95% CI: 0.57–0.80)	71.43% (95% CI: 0.58–0.82)

## Data Availability

The analyzed datasets generated during the study are available from the corresponding author upon request.
